# Construe with metabolic (dysfunction)-associated fatty liver disease, metabolic fatty liver disease, metrics, and contributions

**DOI:** 10.1590/1806-9282.20250709

**Published:** 2026-06-29

**Authors:** Ilker Sengul, Demet Sengul

**Affiliations:** 1 Giresun University, Faculty of Medicine, Division of Endocrine Surgery – Giresun, Turkey.; 2 Giresun University, Faculty of Medicine, Department of General Surgery – Giresun, Turkey.; 3 Giresun University, Faculty of Medicine, Department of Pathology – Giresun, Turkey.

*Mater artium necessitas*. Fatty liver disorders remain a crucial and challenging issue in hepatology^1,2^. We have read with great interest the research article "Metabolic (dysfunction)-associated fatty liver disease metrics and contributions to liver research"^3^. This utilitarian research desiderates investigating and describing novel metabolic fatty liver disease (MAFLD), which sheds light on how this new terminology will affect the scientific community. For this purpose, Suoh et al.^3^ incorporated the English-language literature, adopting MAFLD, published from 2020 to 2023, derived from PubMed, Web of Science, and Scopus indexes by exporting their record files, which had been imported into R (4.4.0) and RStudio (2024.04.2) via the bibliometrix package (4.2.3). The authors have analyzed the publication metrics with publication counts, publishing journals, author countries, author keywords, and citations in order to evaluate the research impact and the relevant key topics. Nevertheless, would any indexing error(s), *per se*, alter the porism of this study? *Inter alia*, this comprehensive analysis targeted publication indicators with a quantitative measure, which demands highlighting the remarkable global research contribution rather than the quality of the included studies in this field. What's more, would emphasizing specific instances of how this modification from the revised MAFLD definition to clinical features affects clinical practice or health care outcomes? As such, this phenomenon requires accelerating basic and clinical research that might lead to flourishing management modalities. This issue merits further investigation. We thank Suoh et al.^3^ for their meritorious study on MAFLD*—post tenebras lux*.

## Data Availability

The datasets generated and/or analyzed during the current study are available from the corresponding author upon reasonable request.
